# Integrated diagnostics: proceedings from the 9th biennial symposium of the International Society for Strategic Studies in Radiology

**DOI:** 10.1007/s00330-012-2510-6

**Published:** 2012-06-15

**Authors:** G. P. Krestin, P. A. Grenier, H. Hricak, V. P. Jackson, P. L. Khong, J. C. Miller, A. Muellner, M. Schwaiger, J. H. Thrall

**Affiliations:** 1 Department of Radiology, Erasmus MC, University Medical Center Rotterdam, Rotterdam, The Netherlands; 2 Service de Radiologie Polyvalente Diagnostique et Interventionnelle, Hôpital Pitié-Salpetrière, Paris 13, France; 3 Department of Radiology, Memorial Sloane-Kettering Cancer Center, New York, NY 10065 USA; 4 Department of Radiology, Indiana University School of Medicine, Indianapolis, IN 46202 USA; 5 Department of Radiology, Queen Mary Hospital, University of Hong Kong, Hong Kong, China; 6 Department of Radiology, Harvard Medical School and Massachusetts General Hospital, Fruit Street, Boston, MA 02114 USA; 7 Nuklearmedizinische Klinik und Poliklinik, Klinikum rechts der Isar der Technischen Universität München, 81675 Munich, Germany

**Keywords:** Radiology, Diagnostic techniques and procedures, Informatics, Algorithms, Efficiency, Organizational

## Abstract

The International Society for Strategic Studies in Radiology held its 9th biennial meeting in August 2011. The focus of the programme was integrated diagnostics and massive computing. Participants discussed the opportunities, challenges, and consequences for the discipline of radiology that will likely arise from the integration of diagnostic technologies. Diagnostic technologies are increasing in scope, including advanced imaging techniques, new molecular imaging agents, and sophisticated point-of-use devices. Advanced information technology (IT), which is increasingly influencing the practice of medicine, will aid clinical communication and the development of “population images” that represent the phenotype of particular diseases, which will aid the development of diagnostic algorithms. Integrated diagnostics offer increased operational efficiency and benefits to patients through quicker and more accurate diagnoses. As physicians with the most expertise in IT, radiologists are well placed to take the lead in introducing IT solutions and cloud computing to promote integrated diagnostics. To achieve this, radiologists must adapt to include quantitative data on biomarkers in their reports. Radiologists must also increase their role as participating physicians, collaborating with other medical specialties, not only to avoid being sidelined by other specialties but also to better prepare as leaders in the selection and sequence of diagnostic procedures.

*Key Points*

• *New diagnostic technologies are yielding unprecedented amounts of diagnostic information*.

• *Advanced IT/cloud computing will aid integration and analysis of diagnostic data*.

• *Better diagnostic algorithms will lead to faster diagnosis and more rapid treatment.*

## Introduction

Making medicine more personalised and precise will entail increasing emphasis on, and precision in, diagnostics. To date, experience and intuition have been physicians’ primary tools for integrating clinical information and test results into a diagnosis. However, new diagnostic technologies, along with high-throughput technologies for biomedical analysis, are now yielding unprecedented amounts of data reflecting not only anatomy and pathology, but molecular processes and genetics as well [1]. Scientists continually report new subtypes as well as new genetic, serum, and tissue biomarkers of disease [1, 2]. With time, data overload is only going to increase [3]. The unassisted human mind cannot make optimal use of this avalanche of information either in the clinic or for research. What is required is a new concept of “integrated diagnostics”: the convergence of imaging, pathology, and laboratory tests with advanced information technology (IT).

Making the most of available data and resources has never been more critical. Around the world, pressures are mounting to expand healthcare coverage while reducing its cost. In the USA, for instance, where millions of individuals remain uninsured, healthcare costs account for nearly $1 of every $5 of national income and are considered a major obstacle to economic recovery and long-term growth [4, 5]. Much of the spending is wasteful—the result of inappropriate or fragmented care, in which diagnostic tests and treatments are underused, overused, or delayed. It is clear that flaws in processes such as communication and coordination [6], as well as gaps in medical knowledge, are obstructing the path to personalised medicine, in which the right treatment is delivered to the right patient at the right time. High rates of medically preventable deaths have been observed in the USA as well as other developed countries, including the UK, Denmark, and Australia [4, 7]. In the USA alone, it is estimated some 40,000 to 80,000 patients die as a result of missed diagnoses while approximately 100,000 die from non-error adverse drug effects each year [8, 9]. More appropriate use of existing diagnostics, as well as the development of better diagnostics, could prevent incorrect and delayed diagnoses and enable more appropriate treatments with lower costs and better outcomes.

Recent studies suggest that integrated delivery systems, in which specialists share information and work in teams to determine which tests and procedures are necessary, result in less costly, higher-quality care [10, 11]. By providing rapid access to patient data and decision support tools, clinical IT can play an essential role in integrating healthcare services [6]. Furthermore, widespread implementation of advanced IT in research and clinical settings could hasten the validation and application of new disease biomarkers and diagnostic algorithms.

This article considers some of the many ways that integrated diagnostics with advanced IT could increase the quality and efficiency of healthcare—and perhaps even facilitate a paradigm shift from curative to preventive medicine. Special attention is given not only to the importance of imaging in unravelling the links between various types of diagnostic data, but to radiologists’ future role in the practice of integrated diagnostics.

## Paradigm-changing diagnostic technologies

The gradual shift toward precision medicine now underway has been driven by rapid growth in diagnostics. Radiology has seen enormous growth in imaging utilisation [12] as well as the development of new imaging techniques that provide functional as well as anatomical information [13]. Automated machines in centralised laboratory facilities turn out ever-increasing amounts of genetic and molecular biomarker data [14, 15], while pathology laboratories perform ever-escalating numbers of semi-automated immunohistochemical assays [15].

In the future, new, easy-to-use, miniaturised diagnostic “lab-on-a-chip” devices that employ nanomaterials, microarrays, and microfluidics will likely cause the volume of diagnostic data to grow even more rapidly [16]. The combination of these miniaturised devices with novel imaging techniques and more conventional diagnostic techniques may change diagnostic paradigms.

### Magnetic nanoparticle-based point-of-care diagnostic technology

Some of the most exciting developments in diagnostic technology include a new class of miniaturised devices that depend on magnetic nanoparticle (MNP) tags carrying antibodies to biomarker proteins [17, 18]. Since biological samples lack any detectable magnetic signal and magnetic properties are not affected by turbidity or sample impurities, such devices have the capacity to be both highly sensitive and highly specific.

Two devices that utilise MNP tags are now available, and both are well suited to point-of-care-diagnostics, as they require no specialised laboratory facilities or sample preparation, are inexpensive to use, and can be employed by personnel with varying levels of education and experience [19, 20]. One of these devices employs giant magnetoresistive (GMR) sensors; it can simultaneously measure the concentration of multiple biomarkers in clinical samples such as urine, serum, cell lysates, or saliva and is 1,000 times as sensitive as the current clinical standard, ELISA [17]. The other such device uses miniaturised nuclear magnetic resonance technology to analyse unprocessed biological samples and detect biomarkers on cells as well as in biological fluids; it can simultaneously and rapidly test for multiple biomarkers [20] with an accuracy that may exceed that of conventional cytology [21]

### Molecular imaging

Over the past decade, molecular imaging has been hailed as the future of radiology [22]. Although most molecular imaging studies are still performed in animals, there is optimism that more of these imaging techniques will soon be adopted clinically. For example, there are new imaging techniques, such as hyperpolarized ^13^C MRI [23], many promising new PET tracers for diseases including cancer and Alzheimer’s disease [24], and some novel multimodal contrast agents [25, 26].

Metabolic imaging with hyperpolarized ^13^C MRI is more than 10,000 times more sensitive than ^1^H magnetic resonance spectroscopy (MRS) and promises to be a valuable clinical technique. The technique has detected changes corresponding to tumour response within 1 day of initiation of treatment and is now being used to explore pyruvate metabolism in human prostate cancer [23]. Hyperpolarized ^13^C-pyruvate MRI could be particularly valuable in the brain, where the rapid rate of glucose metabolism precludes the use of FDG-PET to detect response to treatment [27].

Promising new multimodal contrast agents now under investigation include MRI–Raman–photoacoustic nanoparticles [25] and C-Dots [26]. MRI–Raman–photoacoustic nanoparticles combine sensitivity, specificity, resolution, and depth. Detection of these nanoparticles by MRI is valuable for presurgical planning and intraoperative guidance. The gold layer delivers a photoacoustic signal, which is also useful in intraoperative imaging and amplifies the Raman signal, enabling highly sensitive optical detection of the tumour and its complete removal with clean margins, as has been demonstrated in mouse models [25].

C-Dots, small enough (7 nm) to be cleared by the renal system, are radiolabelled agents functionalised to target specific tumour antigens. Theses agents, detectable with both optical technology and PET and in real time using handheld intraoperative tools, are now in an FDA-approved trial [26].

### Novel imaging techniques

Diagnostic accuracy will also be improved by using acoustic radiation force impulse (ARFI) imaging and by the addition of optics to catheters and needles. ARFI is a recently developed technique that has been shown to aid ultrasound visualization of needle placement, providing accurate positioning that can be used to guide biopsies in interventional radiology [28]. Optical fibres have been developed that are small enough to reach the coronary arteries via catheters to allow direct examination of arteries for atherosclerotic plaque. In animals, these fibres have been employed in catheters to provide co-localised information on microstructure and molecular function in coronary arteries, using near-infrared fluorescence and frequency domain imaging [29], or to visualise bile duct anatomy during surgery [30]. Optical catheters and endoscopes can be used to discriminate between normal, precancerous, and malignant tissue by measuring autofluorescence [31] and could be useful for surgical tumour margin assessment, for biopsy guidance in superficial tumours (such as breast and prostate cancers), and for therapy response monitoring. Other optical catheters use Raman spectroscopy to interrogate arteries, generating an endoluminal map of the deposition of cholesterol, triglycerides, and other plaque components [32]. These technologies, when they become clinically available, will be able to provide real-time diagnostic information [33].

### Future diagnostic algorithms

**Fig. 1:**
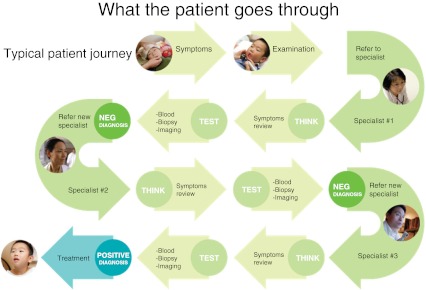
Schematic of the current diagnostic algorithm for many patients. Courtesy of Gene Saragnese, MD, Philips Healthcare, Andover, MA

Not all of the new diagnostic techniques discussed above involve imaging, but many cross the lines between traditional specialties. Optimal employment of these techniques will likely require new paradigms for diagnosis. At present, the typical diagnostic journey (Fig. 1) starts with a referral to a specialist, who reviews the patient’s symptoms and orders one or more tests [34]. If those prove negative, the patient is referred to a second specialist, who again goes through the process of reviewing symptoms and ordering tests. This iterative process may be repeated multiple times before a positive diagnosis is reached. Worse, the patient may be treated without having received an accurate diagnosis, which fails to help the patient and wastes both time and resources.

A future diagnostic algorithm could start in the primary care physician’s office, using a handheld device to measure diagnostic biomarkers for diseases, such as cancer. If the results are positive, the patient could then be sent for molecular whole-body imaging to identify the site(s) of disease. Then after fine-needle biopsy of the tumour, μNMR could be used to identify the presence of specific tumour antigens, which would confirm the diagnosis and guide the selection of treatment. Finally, if surgical resection is appropriate, multimodal contrast agents could be used for presurgical planning and intraoperative imaging to ensure the complete removal of the tumour [25].

The future of molecular imaging and other novel diagnostic techniques will depend on close cooperation among those who develop the techniques, regulatory bodies, and industry and will need wide acceptance by the pharmaceutical industry. Furthermore, radiologists need to be part of a business model, together with others responsible for diagnostics, to drive the success of personalized medicine and integrated diagnostics.

The avalanche of data that existing and new diagnostic technologies generate can provide the information needed for rapid and accurate diagnosis. However, algorithms must be developed that optimize the selection of diagnostic methods and the order in which they are applied to achieve optimal speed and cost efficiency in diagnosis. The accumulated diagnostic data must be synthesised and analysed—a complex and challenging process likely to require collaboration among medical specialists and the implementation of sophisticated IT tools [35].

## The importance of advanced IT for integrated diagnostics

Achieving cost-effective, evidence-based use of diagnostics requires balancing many factors, including the negative and positive predictive values of available tests, the costs and risks associated with them, and the age and clinical history of the individual patient, to name just a few. As more diagnostic tests become available, the difficulty of weighing these factors only grows [36]. Advanced IT tools for data sharing, data mining, statistical modelling, and clinical decision support can not only prevent basic medical errors due to poor communication of information, they can help integrate and analyse complex data to clarify what test should be applied when, based on evidence rather than intuition or habit [36, 37].

Clinical test results make up a clinical phenotype of disease expression, while imaging biomarkers make up an imaging phenotype, and so forth. By finding links between the various phenotypes as well as patient and disease genotypes, it is possible to increase diagnostic speed and precision and unravel biological mechanisms of disease [38]. Thanks to the digitisation of biomedical data, bioinformatics is becoming an increasingly powerful tool for this purpose.

Bioinformatics involves the use of computational techniques in pattern recognition, data mining, machine learning, visualization, and other applications to analyse and identify connections between various types of biomedical data [38]. Computerised data mining can be done in a “structured” fashion, in which the computer searches for features or patterns initially selected by the human operator, or in an “unstructured” fashion, in which the computer identifies patterns on its own. In the structured approach, the human-identified features are subjected to hypothesis testing not by creating new prospective experiments, but by extensively searching data derived from prior experiments [38]. Naturally, the larger the database used, the greater the likelihood of identifying meaningful patterns and correlations although the computing demands are high. Cloud computing may provide a solution.

### Cloud computing

Cloud computing is, essentially, the use of servers on a network to provide rapid, secure, remote access to shared computer resources (data storage, applications, or other computer services) [39, 40]. It allows users to store and utilise much greater volumes of data, at a much lower cost, than they could locally. It also enables them to access their data and applications from anywhere, anytime via the Web. Data can be shared across institutions, platforms, and applications. In addition, cloud computing supports high-performance computing, in which large quantities of data are analysed through parallel processing on multiple computers [41, 42].

Cloud computing is increasingly penetrating every corner of daily life. To find an example of it, one must look no further than the popular messaging and collaboration applications offered by Google, such as Gmail, Google Calendar, and Google Docs. With such applications, users can send e-mail, store, access, and share documents, and update their calendars from stationary computers or mobile handheld devices without purchasing any additional hardware or software [43].

### Health information exchanges

Cloud computing as well as remotely accessible internal organisational networks are increasingly being used to make accessing and integrating medical data easier. For example, at the University of California Los Angeles, the Radiology–Pathology project is developing a concise, electronic Web-based report that combines clinically pertinent imaging and pathologic diagnostic information; physicians will be able to access the report securely through the Web even when they are away from their primary practice locations [44]. In the USA, at least 196 health information exchanges have been created, through which patient data are exchanged between facilities within a hospital system, community, or region [45]. With support from the National Institute of Biomedical Imaging and Bioengineering (NIBIB), the Radiological Society of North America is piloting a patient-centred secure image-sharing network that relies on cloud computing [46]. Furthermore, Google, Microsoft, and several other companies have launched cloud-based personally controlled health records (PCHR) systems [47] that allow patients to store, access, and share their healthcare records on the Web, using password protection comparable to that found on online banking sites. With PCHR systems such as Google Health and Microsoft HealthVault, individuals may grant access to their information to care providers, family members, or others—including researchers [47].

At the most basic level, systems such as health information exchanges and PCHRs facilitate integrated diagnostics by enabling physicians to rapidly assemble patients’ health records and test results, regardless of where or when they were originally obtained. The resulting improvements in communication are expected to reduce medical errors and increase healthcare quality, safety, and efficiency [48].

In the longer term, cloud-based systems for sharing patient data could also have a tremendous impact on research into new diagnostic, treatment, and preventive methods. Today, researchers are usually only able to study patients from their own institutions. However, widespread adoption of PCHRs, for example, could make it possible for researchers to quickly and affordably recruit patients from other academic and community healthcare centres, even from other countries [47]. In addition, research institutions may themselves share large amounts of data through cloud computing. The resulting, potentially “global” study cohorts could dwarf those of even the largest of today’s multicentre studies, dramatically expanding the possibilities for population studies and the power of bioinformatics analyses.

By evaluating large, well-defined population samples over extended periods of time, population studies allow researchers to better separate the effects of environment, lifestyle, genetics, and other factors on disease development and long-term health outcomes. For example, the ongoing Rotterdam Study, initiated at Erasmus University in 1990, assesses factors influencing the development of chronic diseases in a large cohort of subjects enrolled at age 45 years or more; participants are interviewed at baseline and periodic follow-up visits, are examined in the study centre, undergo imaging, and have bodily fluids collected for molecular and genetic analyses [49]. The Rotterdam Study has identified numerous associations between lifestyle, clinical, molecular, and genetic variables and common chronic diseases. In 2005, an MR imaging unit was added to the Rotterdam Study enabling the identification of correlations between imaging biomarkers (imaging phenotypes), clinical phenotypes, and genotypes. Through this process, the value of imaging biomarkers in predicting disease can be established and validated. By 2009, the Rotterdam Study had a population of approximately 15,000 and had resulted in nearly 1,000 research articles and reports [49].

### Population imaging

Bioinformatics is already affecting radiology and will push radiology into becoming more information rich. This transition will accelerate with increasing use of population imaging—the large-scale acquisition and analysis of images in controlled patient cohorts. Population imaging employs computational radiology techniques such as unstructured and structured data mining, image segmentation, and statistical modelling to map and summarise imaging features from large image databases and thus extract meaningful imaging biomarkers [38]. The biomarkers may be anatomic structures, disease manifestations, tumour characteristics, or haemodynamic abnormalities.

The summation of one or more imaging features, or biomarkers, from a global data set can be considered a phenotypic “population image” representing a particular disease or health state. In clinical care or clinical trials, population images may be used as a reference to classify individuals or patient groups into diagnostic categories [38, 50, 51]. Radiologists can play a key leadership role in providing the needed intuition to productively integrate the computational information from population images with personal medical information [38].

Digital imaging techniques have enabled the development of new methods for automating and increasing throughput of image processing, allowing faster, more precise visualisation and quantification of imaging findings and thus facilitating population imaging studies [50, 51]. Though expensive, non-invasive digital MRI is particularly valuable for assessing chronic disease longitudinally, as it can be done serially, starting before the onset of disease, and can provide information on multiple levels, anatomic, metabolic, and functional. As part of the Rotterdam Study [49], population imaging with MRI has been used to examine the causes and consequences of brain changes over time. Using automated image segmentation and other computational radiology techniques, the investigators identified numerous relationships between quantitative and qualitative imaging features and clinical, pathologic, laboratory, and genetic findings related to stroke risk and the development of dementia [50].

These imaging biomarkers may facilitate prediction of future disease onset, the development and implementation of preventive measures, and even the development of preclinical diagnostics. Biomarkers derived from population studies may also be used to detect and characterise clinically apparent disease, guide research into new treatments, and assess treatment responses in clinical trials and practice [50, 52]. Furthermore, because of the statistical power provided by large sample sizes, population studies using global databases could potentially replace individual prospective studies as a means of validating new biomarkers, saving both time and money [38, 51].

Advanced IT is now making unprecedentedly large-scale imaging research feasible. For example, cloud computing, along with high-performance computing, is likely to play an important role in Euro-BioImaging, a pan-European research infrastructure project that is currently in its preparatory phase. The project is expected to offer platforms for storing, remotely accessing, and post-processing biological and medical imaging data on a large scale to enable multinational sharing of imaging data, reuse of existing data in light of new questions, and advanced analysis of imaging data away from its place of origin [53, 54].

### Clinical decision support

Interactive clinical decision support and other computing tools are now available to help both clinicians and patients make optimal use of health information. Through PCHRs, patients may enter their own test results into disease-management tools [47]. According to Clarient (Aliso Viejo, USA, a GE Healthcare company), its service PATHSiTE® allows physicians not only to view high-resolution digital immunohistochemistry slides along with detailed interpretive reports, but also to review and analyse flow cytometry test results, view patients’ complete case histories, and create tumour board presentations via a secure Web portal [55, 56]. In another example, the recently introduced platform Qualibria brings together data from an organization’s other IT platforms, relevant evidence-based best practices, and the organization’s own clinical standards in a single display, according to GE Healthcare (Waukesha, WI, USA) [56, 57]. Alerts are automatically issued when variations from desired outcomes are detected. Using the platform, organizations can share information and incorporate each other’s best practices [57].

Prediction models for assessing pre- and post-test probabilities of disease are another form of clinical decision support [37, 58, 59]. Such models can be used to tailor diagnostic algorithms to individual patients, first by determining if a particular test is likely to be of value, and then by incorporating the test result with other variables to assess the likelihood of the presence, or future development, of disease [37]. These models, however, are setting specific and do not necessarily remain applicable as populations change [59, 60]. Theoretically, using high-performance computing in a cloud, the models, some of which are already on the Internet, could be automatically updated for specific settings in real time, whenever new patient data are entered into electronic medical records systems [37, 58].

### Concerns and challenges

Realizing the potential of advanced IT for improving diagnostics essentially involves three steps: (1) digitizing medical information and establishing connectivity so that data can be rapidly accessed and shared; (2) implementing advanced analytics techniques across data types to change information overload into insights; and (3) integrating these insights into healthcare workflow to inform decisions [56]. While other industries, such as banking, have learned to use advanced IT to aggregate, integrate, and apply large amounts of digitized data, healthcare is mainly still working to achieve digitization and connectivity. This is by far the most financially and culturally challenging stage, and the benefits will increase with each successive step [56].

Costs are one of the major obstacles to building and maintaining the infrastructure for advanced IT. Achieving digitization and connectivity is expensive, though providers, payers, and patients benefit from the resulting increases in efficiency [48]. As noted earlier, facilities may minimise IT costs by sharing infrastructure and other computer resources through cloud computing. Until recently, health information exchanges generally relied on centralized management of data-sharing agreements between individual facilities or healthcare networks by independent health information organizations, but “in-house” development of exchange capabilities is now on the rise [45, 47]. Health information exchanges in the USA have mainly been funded by grants and contributions from participating facilities, and few have become self-sustaining [45, 48]. Notably, of the more successful ones, most provide not only data exchange, but additional tools such as advanced analytics, quality reporting, or clinical decision support [45]. There is some debate as to whether capabilities for electronic exchange of health information should be funded by those who use them or, as is the case in the UK, by the government [48]. With PCHR systems, compared with conventional health information exchanges, many expenses—along with control of patient data—are shifted away from healthcare organizations [47].

Concerns about patient privacy and data security are another considerable hurdle to large-scale data integration. Doubts about the security of cloud computing linger, and some healthcare organizations still prefer to maintain responsibility for data security by containing their patient data within their own firewalls. In the USA, laws regarding patient privacy and secure handling of patient information have not been harmonized across states, and standards have yet to be defined for locating and matching patient information across healthcare facilities and networks [61]. Particularly with PCHR systems, data may be shared, wittingly or unwittingly, with entities not bound by HIPAA regulations [47]. Even when Internet portals are password protected, sharing of patient data with multiple parties may increase the risk of accidental or deliberate security breaches. Notably, the Euro-BioImaging infrastructure project is expected to provide European standards not only for imaging data storage and analysis, but for data protection [53].

Finally, particularly when conducting “global” studies using advanced IT, making sure that data have been collected and entered consistently, using standardised nomenclature, is a great challenge. Apart from the fact that testing protocols, technology, and diagnostic accuracy typically vary from one setting to another, diagnostic terminology varies among diagnostic disciplines and within and between institutions. Tools such as natural language processing can be used to rapidly analyse aspects of unstructured reports [62]. However, ultimately, the goal should be widespread implementation of structured reporting with standardised terminology. In 2007, to work toward this goal, nine countries came together to found the non-profit International Health Terminology Standards Development Organization (IHTSD) [63]. Its mission is to acquire, develop, maintain, promote, and enable the uptake and correct use of standard healthcare terminology products around the world [63]. Among other terminologies, the organization administers the rights to the Systemized Nomenclature of Medicine Clinical Terms (SNOMED-CT), which is widely considered the most comprehensive, machine-readable clinical vocabulary available [63, 64]. The IHTSD has now expanded to include seven other countries. Membership fees are adjusted on the basis of national income [63].

## The role of radiologists

Integrated diagnostics presents an opportunity for radiology to evolve as a profession and deepen the impact of the radiologist on healthcare [34]. Imaging plays a central role in diagnostics because of its ability to visualize disease in vivo, to measure its extent and severity anatomically, and to characterize it functionally and metabolically both in the context of initial work-up and in response to therapy. As the practice of medicine moves away from an intuitive, experience-based model to empirical, evidence-based and to personalised medicine, diagnostic precision becomes paramount in order to select the particular treatment that will best help each individual patient [36].

Diagnoses depend on multiple components that include not only imaging, but also clinical observation, pathology, laboratory, and genomic tests. To date, there is too little coordination between the medical specialties responsible for ordering and performing these tests, nor is there enough consideration as to the optimal order of tests. This will change in a world of integrated diagnostics, where, instead of relying on individual provider bias in the selection of tests, data from diverse sources will be used to determine the most efficient diagnostic algorithms. Imaging will be incorporated judiciously into these integrated diagnostic algorithms, complementing other diagnostic techniques in order to maximise efficiency and minimise waste. This may satisfy payers who currently perceive radiology as a major contributor to cost escalation. The American College of Radiology (ACR) has taken some initial steps towards the development of integrated diagnostics in its Appropriateness Criteria® [65], which are developed by panels of radiologists and other specialists, by including clinical symptoms and the results of laboratory tests in determining imaging appropriateness. Other guidelines have been prepared in other countries, notably in the UK by the Royal College of Radiologists and France by the Société Française de Radiologie; the European Commission is currently revising their guidelines in conjunction with the European Society of Radiology.

Ideal integrated diagnostic algorithms will depend on the acquisition of sequential information for more accurate and specific diagnoses. Algorithms should start with the least expensive tests that will narrow down the possibilities and effectively increase the positive predictive value of subsequent tests [36]. Each test in a diagnostic algorithm should break the tested population into smaller subpopulations with specific phenotypes, or sets of observable traits, for which the associated risks of disease are better defined. As medical knowledge increases, so, too, do the numbers of disease phenotypes—and genotypes—that must be ruled out or in; this adds difficulty to the diagnostic process but makes patient care more efficient by helping to determine which treatment, if any, is likely to work. For example, the number of types of lymphoma recognised has exploded from 5 to 53, and the type drives the choice of treatment [36].

Once developed, application of integrated diagnostic algorithms should result in faster diagnoses, with greater precision and less cost. As an example, we can consider the algorithm for the diagnosis of patients presenting to emergency departments with chest pain. In the current standard of care in the USA, all patients with suspected acute coronary syndrome (ACS) are first given an electrocardiogram to detect arrhythmias and a troponin assay. Those with ST segment elevation myocardial infarction (STEMI) are immediately recognised and treated. But the majority of patients, who do not have STEMI, are given presumptive therapy with aspirin, oxygen, and β-blockers and are evaluated with serial electrocardiograms and cardiac enzyme assays over a period of 8 to 12 h. If troponin levels do not rise, rest and/or stress-imaging studies are performed to rule out ischaemia. While this process has lowered missed diagnoses of ACS, it is both time-consuming and expensive (emergency evaluations for acute chest pain cost approximately $10 billion annually in the USA) [66].

However, this diagnostic algorithm for suspected ACS may soon change as recent studies have explored the integration of non-invasive coronary CT angiography (CCTA) into the algorithm to rule out ACS or severe stenosis early on in the work-up, for example, after just one or two enzyme assays [66, 67]. Even though CT examinations are expensive, these studies have found that the use of CCTA is cost effective because it reduces time to diagnosis and the number of hospital admissions. With CCTA, the median time to diagnosis fell from 15 h to just 3.4 h in one study [66]. The main strength of CCTA is its high negative predictive value, which allowed as many as 67 % of those imaged to be discharged while the remainder were divided into distinct higher-risk categories suitable for further testing [66, 67]. Moreover, CCTA lowers hospital readmission rates without causing adverse cardiovascular events in the near or mid term, with readmission rates falling from 9 % to 1 % in one study [67].

In the development of new integrated diagnostic algorithms, the sequence of tests should be determined by multidisciplinary teams of physicians that include radiologists and other specialists that care for particular patient populations. Determining the optimal use of diagnostic tests will require heavy use of IT to calculate the probabilities and discern linkages between data and disease, because the complexity of the data precludes depending solely on intuition and experience. It will also be essential to be able to share information among disciplines, which will require the adoption of standard nomenclature, using ontologies such as SNOMED [64] and RADLEX [68]. Adopting a common language will be a major hurdle but will make it possible to use natural language processing to extract diagnostic phenotypes from unstructured reports [62], better understand the pattern and progress of disease, and facilitate the development of reproducible algorithms, decision trees, and data assembly tools to integrate data from diverse sources.

In order to be effective in a world of integrated diagnostics, radiologists will need to be able to move beyond their analogue world of qualitative interpretation of radiological signs, non-specific tissue contrast, and free text reports—descriptive findings that may not interpret the study in light of the clinical setting. Radiologists must think in terms of quantitative data on imaging biomarkers as well as quantitative data from pathology tests and clinical information. Such quantitative data will be necessary to develop and apply diagnostic algorithms with decision support tools. The Quantitative Imaging Biomarker Alliance (QIBA), launched in 2007 and whose membership includes radiology researchers, industry, and government regulators, has made some progress in regard to the development of quantitative imaging biomarkers, the development of uniform protocols for imaging in clinical trials, and education at society meetings [69, 70].

Achieving reliable, reproducible, and comparable quantitative imaging results amongst different institutions will require standardization of imaging equipment across vendors, standardization of imaging protocols, and correlations with clinically meaningful metrics. Equipment manufacturers must be convinced of the necessity to standardize. Radiology societies will need to educate their memberships by including quantitative imaging presentations at national meetings, as well as exhibits related to software that can be incorporated into routine radiologic practice. Moreover, imaging biomarkers will need to be accepted by regulators in order to foster their acceptance. We can anticipate resistance, both from vendors who want to be seen as different from their competitors and from radiologists who fear losing value due to automation [70]. Most importantly, a major effort will be necessary to set up standardized reporting for all clinical fields of imaging and to train radiologists to provide clinicians with standardised reports.

More radiology training and subspecialisation will also be helpful in a world of integrated diagnostics. Benefits of subspecialisation include higher level of expertise, higher quality of patient services, improved efficiency, and improved interaction and common language with other clinicians. Subspecialization improves the standing of radiology as a specialty, enriches radiologists’ armamentarium in turf battles, provides a stronger basis for clinical research, and supports recruitment of students into radiology. However, subspecialization needs to evolve from one-dimensional organ/system-based models to three-dimensional patient-centred matrix models that consider the disease and the presenting clinical problem [71]. Training models need to include complex tasks for radiologists, including tumour boards, interdisciplinary conferences, and new models of patient-centred care in oncology, diabetes, and metabolic disorders, among others.

Revised subspecialty categorisation is also needed in order to reflect the continuing evolution of radiology and the development of hybrid imaging methods, such as PET/CT and MR/PET and novel molecular tracers, and to acknowledge that no one can master the entire field of imaging [70]. Hybrid imaging technologies require physician expertise in both functional and anatomic imaging and are driving the convergence of radiology and nuclear medicine into the new subspecialty of molecular imaging [72]. In Europe, the European Association of Nuclear Medicine and the European Society of Radiology have recognised the importance of coordinating working practices between radiology and nuclear medicine for multimodal imaging [73]. In the USA, the ACR and the Society of Nuclear Medicine convened the Task Force on Nuclear Medicine Training to define the issues and develop recommendations for resident training [74]. Despite these efforts, it must be recognised that there are a number of institutional barriers that need to be overcome before molecular imaging becomes a subspecialty, such as regulation review requirements, reimbursement, hospital organization, and limitations in the development and cost-efficient supply of novel molecular tracers [73].

Professionally, becoming members of integrated diagnostic and therapeutic teams will benefit radiologists who, in present practice, are nearly invisible, leaving the public largely ignorant of what they do [22, 75]. Radiologists must become more visible, remain physicians, and go for the diagnostic driver’s seat. The current radiology business model is a professional service that is being deconstructed into components by virtue of digitalization, with each component being transformed by computation and by bioinformatics. It has been suggested that a continuation of the present emphasis on image interpretation and reporting, rather than consultation and problem-solving, runs the risk that the radiology profession will become marginalized and commoditized. On the other hand, applying knowledge and wisdom to consultations with referring physicians is unlikely ever to be commoditised [76]. Therefore, in order to retain their standing, radiologists have to recognize that radiology is more than just imaging and engage more directly with both patients and referring physicians.

By becoming more visible to patients and more substantively involved in patients’ medical care as active team members, radiologists will be better prepared for the anticipated future, when they will be actively involved in selecting the sequence of diagnostic procedures. Ultimately, they may even become leaders and doorkeepers routinely in contact with patients [77] in departments of diagnostic medicine that include laboratory science, pathology, and radiology.

## Abbreviations

ACR: American College of Radiology
ACS: acute coronary syndrome
ATFI: acoustic radiation force impulse
CCTA: cardiac computed tomographic angiography
ELISA: enzyme-linked immunosorbent assay
HIPAA: Health Insurance Portability and Accountability Act
GMR: giant magnetoresistive
IHTSD: International Health Terminology Standards Development Organization
ISSSR: International Society for Strategic Studies in Radiology
IT: information technology
MNP: magnetic nanoparticle
NIBIB: National Institute of Biomedical Imaging and Bioengineering
PCHR: personally controlled health records
QIBA: Quantitative Imaging Biomarker Alliance
SNOMED-CT: Systemized Nomenclature of Medicine Clinical Terms
STEMI: ST segment elevation myocardial infarction
